# Study of the Aid Effect of CuO-TiO_2_-Nb_2_O_5_ on the Dielectric and Structural Properties of Alumina Ceramics

**DOI:** 10.3390/ma16145018

**Published:** 2023-07-15

**Authors:** Rafael I. Shakirzyanov, Natalia O. Volodina, Kayrat K. Kadyrzhanov, Artem L. Kozlovskiy, Dmitriy I. Shlimas, Gulzada A. Baimbetova, Daryn B. Borgekov, Maxim V. Zdorovets

**Affiliations:** 1 Engineering Profile Laboratory, L.N. Gumilyov Eurasian National University, Satpayev St., Astana 010008, Kazakhstanshlimas@mail.ru (D.I.S.);; 2 Department of General Physics, Satbayev University, Almaty 050032, Kazakhstan; 3 Department of Science, Abai Kazakh National Pedagogical University, Almaty 050010, Kazakhstan; 4 Laboratory of Solid State Physics, The Institute of Nuclear Physics, Almaty 050032, Kazakhstan

**Keywords:** alumina ceramics, dielectric permittivity, hardening, the effect of the triple additive, perovskite phase

## Abstract

The aim of this work is to study the structural, dielectric, and mechanical properties of aluminum oxide ceramics with the triple sintering additive 4CuO-TiO_2_-2Nb_2_O_5_. With an increase in sintering temperature from 1050 to 1500 °C, the average grain size and the microhardness value at a load of 100 N (HV0.1) increased with increasing density. It has been shown that at a sintering temperature of 1300 °C, the addition of a 4CuO-TiO_2_-2Nb_2_O_5_ additive increases the low-frequency permittivity (2–500 Hz) in alumina ceramic by more than an order of magnitude due to the presence of a quadruple perovskite phase. At the same time, the density of such ceramics reached 89% of the theoretical density of α-Al_2_O_3_, and the microhardness value HV0.1 was 1344. It was observed that the introduction of 5 wt.% 4CuO-TiO_2_-2Nb_2_O_5_ in the raw mixture remarkably increases values of shrinkage and density of sintered ceramics. Overall, the results of this work confirmed that introducing the 4CuO-TiO_2_-2Nb_2_O_5_ sintering additive in the standard solid-phase ceramics route can significantly reduce the processing temperature of alumina ceramics, even when micron-sized powders are used as a starting material. The obtained samples demonstrated the potential of α-Al_2_O_3_ with the triple additive in such applications as electronics, microwave technology, and nuclear power engineering.

## 1. Introduction

Ceramic materials based on aluminum oxide (Al_2_O_3_) are widely used in modern science, technology, and industry [1,2,3]. Al_2_O_3_ ceramics are relatively inexpensive components for the creation of refractory materials, wear-resistant parts, electrical insulators, materials for mechanical processing, substrate materials for radio electronics, and microwave technology [4]. In addition, aluminum oxide is a biocompatible material that can be used in medicine for implants. Corundum ceramics have such advantageous properties as a high melting point (2054 °C), compressive strength (up to 3100 MPa), microhardness (5–22 GPa), breakdown voltage (13 kV/mm), electrical resistivity (10^12^–10^18^ Ω∙cm), and thermal conductivity (30 W/m∙K) [5]. Much attention among researchers of ceramic materials is paid to various sintering methods: standard sintering (solid-phase synthesis) in the air or a special atmosphere, plasma sintering, sintering using microwave radiation [6], hot pressing [7], sintering under high pressure [8], spark plasma sintering [9], hot isostatic pressing, etc. [10]. Conventional sintering in the air remains one of the simplest methods since it does not require special equipment other than a muffle furnace with resistance heaters. The main problem with conventional sintering is the high energy consumption due to the high temperatures and process time. For sintering high-density alumina ceramics without special additives, standard ceramic technology requires sintering temperatures higher than 1500 °C [11,12]. Reducing the sintering temperature is possible when using additives for sintering. In this case, compounds with a low eutectic point or compounds that transform into the glass phase during sintering are added to the raw mixture. The additive can either fasten individual particles of the charge by flowing into voids in a softened state or form conditions for solid-phase physicochemical reactions with increased diffusion (both due to the formation of a liquid phase and a defective structure). These features of the sintering process with additives can significantly reduce the sintering temperature of ceramics.

To reduce the sintering temperature and time and obtain a denser microstructure, oxides such as CuO, TiO_2_, MnO, TiO_2_, and MgO are introduced into a raw mixture for aluminum oxide ceramics synthesis [12,13,14]. In some cases, these oxides form eutectic transitions with temperatures below 1500 °C, which reduces the sintering temperature of alumina ceramics. However, despite the large number of works devoted to the synthesis of polycrystalline ceramics from aluminum oxide, an urgent task is to find the optimal compositions of sintering additives that lower the synthesis temperatures while maintaining the functional properties of the final product. Along with the chemical composition of the additive, the granulometric composition of the raw mixture also plays an important role. It is believed that the small particle size of the powder determines the high physical and chemical activity during solid-phase sintering [15]. Previously, in [12], the influence on the final density and temperature of sintering was studied by introducing double additives MnO-TiO_2_ and Cu_2_O-TiO_2_. It was found that introducing combinations (up to 4 wt.%) of oxides is more efficient for obtaining dense ceramics than adding MnO, TiO_2_, and Cu_2_O separately. However, this study was focused on ceramics density measurements and did not include the structural, mechanical, or dielectric properties of the experimental samples. Because of this, the mechanisms of densification were not specified. It was shown in [16] that the introduction of a multi-component additive into the starting submicron alumina (average particle size 0.2 μm) of the composition 9% CuO + 0.9% 2 + 0.1% B_2_O_3_ + 0.1% MgO (additive concentration in the charge was more than 2 mol%), allows obtaining alumina ceramics with a relative density of 99.3% at a sintering temperature of 1070 °C. The introduction of the additive was carried out through a wet dispersion process in a solution of salts of Cu, Mg, Ti, and boric acid. The use of B_2_O_3_ and MgO was associated with intensifying the sintering process and increasing the uniformity of the microstructure. The best results were achieved in the series of samples with the route of double grinding the charge after preliminary calcination. These results demonstrated that dense alumina ceramics can be synthesized at significantly lower temperatures, but the processing route includes wet chemistry, which complicates the production of final products. Another issue that should be pointed out is the absence of phase identification and element distribution in the obtained samples. Yang et al. succeeded in achieving the lowest sintering temperatures compared to the previous work when studying the effect of the ternary addition of 4CuO-TiO_2_-2Nb_2_O_5_ (CTN) to submicron Al_2_O_3_ powder on the sintering of LTCC-ceramics [13]. Mixing the initial powder with 5 wt.% CTN makes it possible to lower the sintering temperature of dense ceramics down to 975° while obtaining excellent microwave dielectric properties, high thermal conductivity, and bending strength. Also, when using the CTN triple oxide additive, it is possible to significantly change the electrical properties of Al_2_O_3_ ceramics and increase thermal conductivity compared to LTCC with composition glass—α-Al_2_O_3_. However, using submicron alumina powder as a starting material can increase production costs. It was demonstrated in [17] that the inclusion of conducting indium-tin oxide in the composition of alumina ceramics creates percolation electrical channels that increase the DC conductivity from 10^−16^ S/cm to 10^−5^ S/cm at concentrations of 0.5–1 mol%. After thermal annealing, the ternary CTN oxide acquires the crystal structure of the quadruple perovskite Cu_3.21_Ti_1.16_Nb_2.63_O_12_, which has a relatively high electrical conductivity value of 1–8 × 10^−2^ S/cm. Thus, obtaining a quadruple perovskite phase in the composition of alumina ceramics can decrease the sintering temperature and change the thermoelectric and electrical properties. Previous studies have achieved similar effects using submicron α-Al_2_O_3_ particles, but the use of such particles increases the cost of ceramic production. To avoid using submicron or nanoparticles as starting powders and understand the effect of the triple additive 4CuO-TiO_2_-2Nb_2_O_5_ on the dielectric, structural, and mechanical properties of aluminum oxide ceramics, further investigation should be done. In particular, micron-sized powders and standard solid-phase ceramic routes, including compaction with binder, must be investigated. The importance of experimental work connected with Al_2_O_3_ sintering is dictated by the crucial influence of processing parameters on the physical properties of the final product [18,19]. In this paper, we study the effect of 4CuO-TiO_2_-2Nb_2_O_5_ on the dielectric and structural properties of alumina ceramics obtained by standard ceramic technology with annealing in a muffle furnace in the air using micron powders of α-Al_2_O_3_. The results of this work revealed some important aspects of the microstructure, phase formation, microhardness, and dielectric properties of alumina ceramics, which are in demand in large-scale production.

## 2. Experimental Part

**Figure 1:**
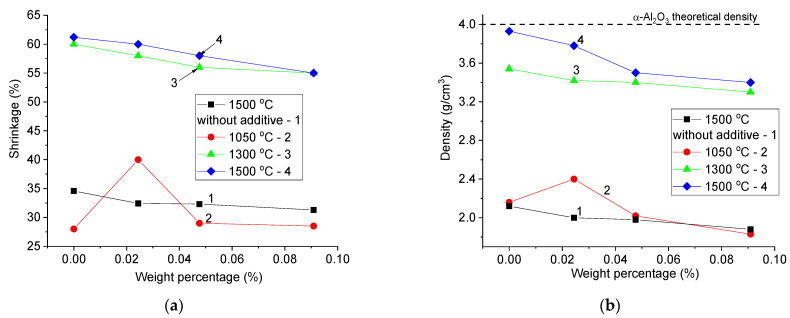
The dependence of volumetric shrinkage (**a**) and apparent density (**b**) of samples on the weight percentage of PVA. 1—sample without addition of CTN and T_s_ = 1500 °C (black squares), 2—sample with addition of CTN and T_s_ = 1050 °C (red circles), 3—sample with addition of CTN, and T_s_ = 1300 °C (green triangle), 4—sample with addition of CTN and T_s_ = 1500 °C (blue rhombs).

Experimental samples were obtained by conventional ceramic technology, including the following operations. Chemically pure reagents produced by Sigma–Aldrich (St. Louis, MO, USA) Al_2_O_3_, TiO_2_, and Nb_2_O_5_ after weighing were mixed in a Fritsch Pulverisette 6 planetary mill. A sintering aid CTN of 5 wt.% CuO:TiO_2_:Nb_2_O_5_ (4:1:2 mole fractions) was mixed with Al_2_O_3_ micron-sized powder (average diameter less than 5 µm) by wet grinding in ethanol. Wet mixing was carried out for 30 min at a rate of 250 rpm using a jar and grinding balls made from wolfram carbide. After that, the powders were dried and re-mixed for 20 min in the same unit. The final drying was carried out at 55 °C for 3 h. The obtained powder was pressed into tablets with a diameter of 12.1 mm and a thickness of 1.5 mm. The maximum pressure during compaction was 200 MPa. In a series of samples, a polyvinyl alcohol (PVA) solution with water (10 wt.%) was used as a binder. The pellets were sintered in a muffle furnace with a resistive heater at temperatures T_s_ = 1050, 1300, and 1500 °C for 5 h. The annealing regime included three steps: in the first step, green tablets were annealed at 300 °C for 2 h to burn out the organic binder; in the second step, the temperature increased to T_s_ with a heating rate of 10 °C/min; and in the third step, T_s_ was maintained for 5 h. After that, the sintered samples were naturally cooled to room temperature for 24 h inside the muffle. The selected sintering temperatures were determined by the result of the analysis of the literature data. For sintered tablets, the apparent density was determined, and the volumetric shrinkage of the tablets was calculated by a formula (1 − V_after_/V_before_) × 100%. The results of volumetric shrinkage and density are shown in Figure 1. As can be seen from the figure, the introduction of a PVA binder reduces the density of sintered tablets and the value of shrinkage. Further, samples with the highest values of apparent densities for each sintering temperature were selected for characterization. For comparison, alumina ceramics were synthesized at the temperature T_s_ = 1500 °C without the addition of CTN. In the case of ceramics synthesized in this work from micron powders, a sharp increase in shrinkage and density is observed in the temperature range of 1050–1300 °C. It can be concluded that in the case of micron powders, the sintering process begins when the CTN additive transforms into a liquid.

Powder diffraction patterns in the range of 2θ = 20–80° were recorded on a Bruker D8 Advance diffractometer (Bruker GmbH, Mannheim, Germany) with CuKα radiation with a wavelength of λ = 0.15406 nm and a scanning rate of 1.8°/min. Scanning electron microscopy (SEM) images and chemical analysis were taken on a Phenom ProX G6 microscope (ThermoFisher scientific, Eindhoven, The Netherlands) at an accelerating voltage of 15 kV with an energy-dispersive analysis (EDX) attachment. The average elemental composition of each sample was evaluated by measuring EDX spectra at a certain number of points (10–15) on the cross-sectional surface. The frequency spectra of the impedance, capacitance, and dielectric loss tangent tan δ at room temperature were recorded using a HIOKI IM3533-01 RLC meter (Hioki E.E Corporation, Singapore) in the frequency range 2–2 × 10^5^ Hz. Before measurements, a conductive silver paste was applied to both sides of the tablet, followed by drying in the oven for 24 h at 60 °C. The conversion of electrical capacitance to permittivity ε’ was performed using the flat capacitor formula. Microhardness HV0.1 was measured on ground samples by the Vickers method with a load of 0.1 kN on a MIKON Duroline-M1 (METKON instruments, Bursa, Turkey) microhardness tester.

## 3. Results and Discussion

**Figure 2:**
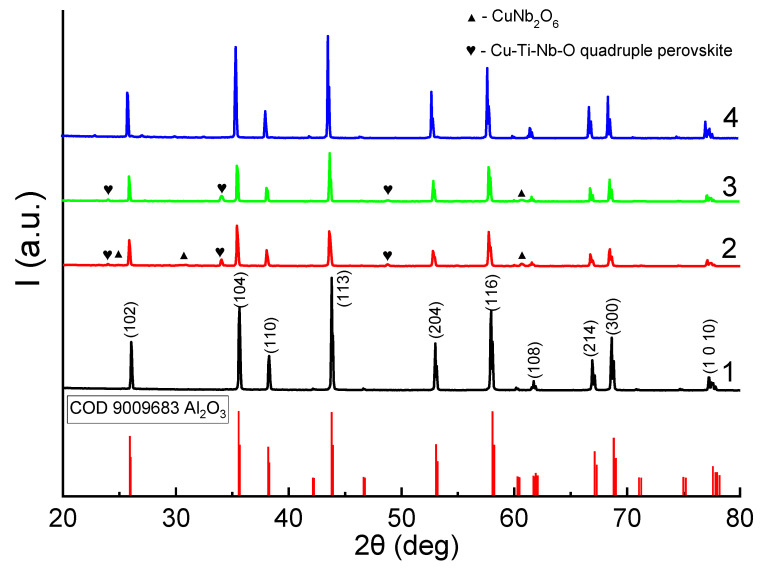
The results of XRD phase analysis of obtained samples. 1—sample without addition of CTN, T_s_ = 1500 °C; 2—sample with additive T_s_ = 1050 °C; 3—sample with additive T_s_ = 1300 °C; 4—sample with additive T_s_ = 1500 °C.

Figure 2 shows the diffraction patterns of the synthesized samples with the highest density and shrinkage, obtained without using an aqueous solution of PVA for the sintering of green tablets. X-ray phase analysis revealed that the main phase in all synthesized samples is α-Al_2_O_3_ (space group R3c), and the secondary phases are CuNb_2_O_6_ (space group Pbcn symmetry) and double-perovskite Cu-Ti-Nb-O (chemical formula Cu_3.21_Ti_1.16_Nb_2.63_O_12_, space group Im-3 [20]). For a sample with a sintering temperature of 1500 °C and the addition of CuO-TiO_2_-Nb_2_O_5_, the peaks of secondary phases were not identified. The mechanisms of formation of alumina ceramics with CTN and phase formation were studied in detail in [21]. According to the results of XRD analysis and TGA analysis, the formation of the A′A″_3_B_4_O_12_ quadrupole perovskite phase at the selected ratio 4CuO-TiO_2_-2Nb_2_O_5_ begins at a temperature of 900 °C through the following phase transformations: Nb_2_O_5_ + TiO_2_ + CuO → CuNb_2_O_6_ + CuTi_2_Nb_2_O_10_ → Cu_3.21_Ti_1.16_Nb_2.63_O_12_. However, at a CTN/Al_2_O_3_ additive ratio of 50/50 wt.%, a shift in the melting temperature to the region of high temperatures is observed. Nevertheless, the main mechanism of compaction of Al_2_O_3_ ceramics is associated not with the transition of the additive to the liquid state but with the solid-state reaction with the formation of the Cu_3.21_Ti_1.16_Nb_2.63_O_12_ phase. This was found for ceramics synthesized from submicron Al_2_O_3_. In the case of the samples studied in this work, it can be concluded that a sharp increase in volume shrinkage and density is shifted to the temperature range of 1050–1300 °C. This suggests that in the case of micron powders, sintering occurs when the CTN additive transforms into a liquid state.

**Figure 3:**
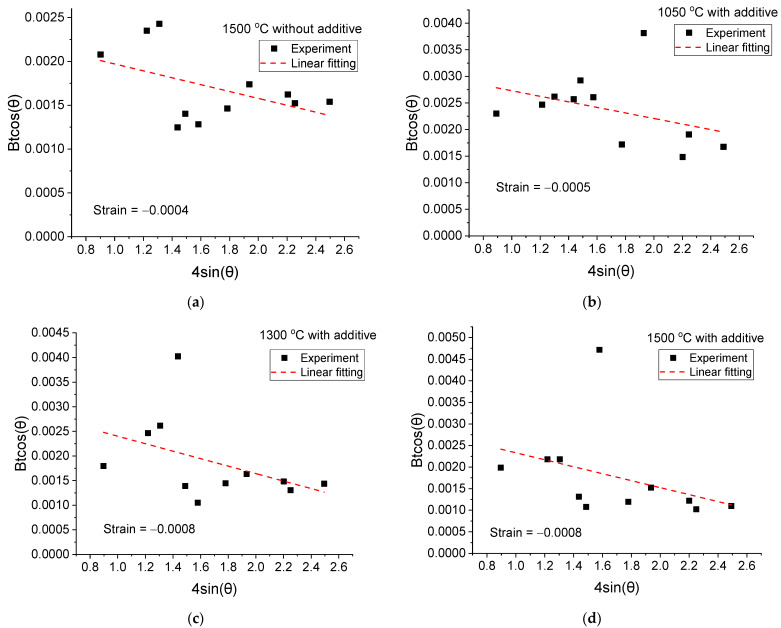
Williamson–Hall plots for α-Al_2_O_3_ phase.

Since the phase composition of ceramics sintered at 1500 °C could not be established, X-ray diffraction analysis of the α-Al_2_O_3_ phase was carried out using reflections with pronounced intensities (102), (104), (110), (113), (204), (116), (214), and (300). The selected reflections were fitted in the Match!3 program to refine the parameters of the peak center and FWHM—B, as well as to determine the parameters a and c of the hexagonal lattice. According to the Williamson–Hall method [22], the stresses in the crystal lattice of Al_2_O_3_ were determined by the broadening of reflections. The results are shown in Figure 3 and Table 1. After fitting the experimental data, it can be seen that the slope of the straight line is negative, which indicates compressive stresses in the crystal lattice of the corundum. The highest stress values were determined in samples with CTN addition and annealing temperatures of 1300 and 1500 °C. In this case, the lattice parameters changed insignificantly for all the samples under study. It is also worth noting that the sample obtained during sintering without the CTN additive is characterized by the lowest stress. Thus, the addition of CTN increases the stresses in the lattice after significant densification during sintering.

**Figure 4:**
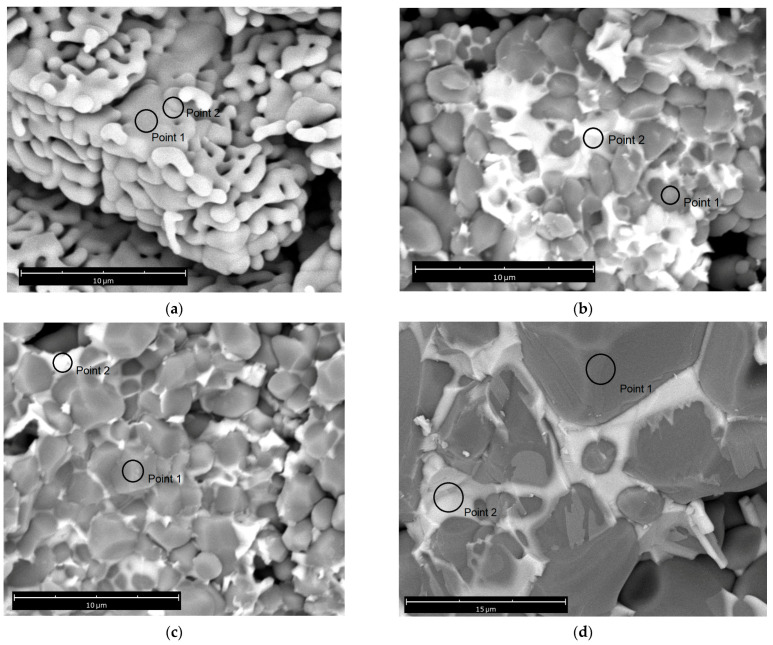
Micrographs of cross-sections of synthesized samples. Points 1 and 2 are spots where EDX spectra were collected. (**a**)—sample without additive, T_s_ = 1500 °C; (**b**)—sample with CTN additive T_s_ = 1050 °C; (**c**)—sample with CTN additive T_s_ = 1300 °C; (**d**)—sample with CTN additive T_s_ = 1500 °C.

**Figure 5:**
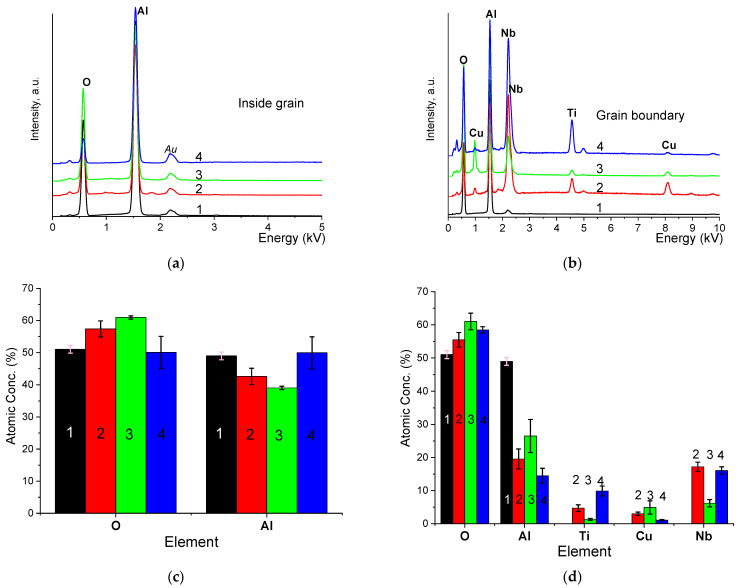
EDX spectra inside the grain (**a**), in the grain boundary (**b**), and the average elemental composition of the grain (**c**), grain boundary (**d**). 1—sample without addition of CTN and T_s_ = 1500 °C, 2—sample with addition of CTN and T_s_ = 1050 °C, 3—sample with addition of CTN and T_s_ = 1300 °C, 4—sample with addition of CTN and T_s_ = 1500 °C.

Figure 4 shows SEM micrographs of cross-sections of tablets for samples with CTN additive and temperatures T_s_ = 1050, 1300, and 1500 °C, as well as for a sample without additive and T_s_ = 1500 °C. For a sample without the additive, high porosity is observed, and the elongated grain size indicates the initiation of coalescence of individual particles without transition to monolithic grains [23]. Cross-sectional images with the additive show two phases due to high contrast. The darker areas (grains) belong to the α-Al_2_O_3_ phase, and the light areas (grain boundaries) belong to the CTN additive containing elements with a large atomic weight of Nb, Cu. The average grain size from SEM micrographs was determined by the intercept line method. It can be seen that with an increase in the sintering temperature in ceramics with the addition of CTN, the average grain size (d_av_) increases, and at the highest temperature, d_av_ increased by a factor of ~5. In addition, porosity is reduced, and the distribution of the intergranular phase becomes more homogeneous. The chemical composition of structural elements was determined by the EDX method (Figure 5) from points in grains and grain boundaries. For example, Figure 4 shows where the elemental composition was studied (point 1—grain, point 2—grain boundary). EDX spectra (Figure 5a) inside the grains show the presence of only Al and O, which indicates the absence of diffusion in Nb, Cu, and Ti inside the grains in the temperature range of 1050–1500 °C. In the case of grain boundaries, the EDX spectra (Figure 5b) contain Al atoms. The content of elements in atomic percentages is shown in Figure 5c,d. It should be noted that the ratio of concentrations C of chemical elements in grain boundaries for a sample with T_s_ = 1300 °C corresponds to the inequality C(O) > C(Al) > C(Nb) > C(Cu) > C(Ti). This confirms the predominant formation of the quaternary perovskite phase since it corresponds to the content of elements according to the chemical formula CTN given earlier. However, the atomic concentrations of C(Nb) and C (Ti) were found to be smaller than in other samples. The possible reason for this is that grain boundary thickness is the smallest in a sample with T_s_ = 1300 °C. Thus, the electron beam of the SEM captures the grain area, and the peaks of Nb, Cu, and Ti were decreased at EDX spectra. The high content of Al in grain boundary can be due to two reasons: (1) the presence of phases with an aluminum content (for example, CuAl_2_O_4_) in a small amount, which is insufficient for determination by X-ray phase analysis [24], or (2) the formation of a solid solution of α-Al_2_O_3_—quadruple perovskite (CTN) [21].

**Figure 6:**
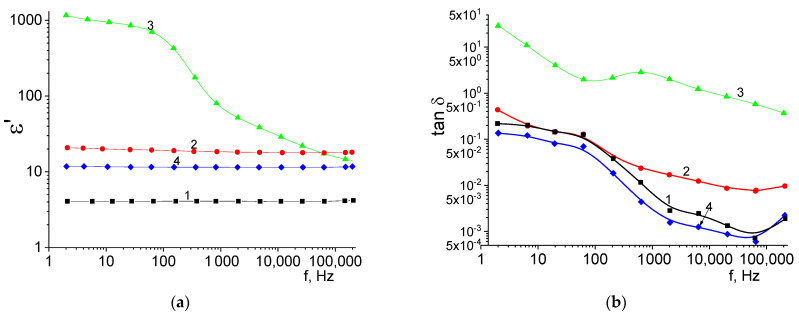
Frequency dependences of permittivity (**a**) and dielectric loss tangent (**b**). 1—sample without addition of CTN and T_s_ = 1500 °C, 2—sample with addition of CTN and T_s_ = 1050 °C, 3—sample with addition of CTN and T_s_ = 1300 °C, 4—sample with addition of CTN and T_s_ = 1500 °C.

The frequency dependences of the permittivity and the tangent of the dielectric loss angle are shown in Figure 6. For samples with annealing temperatures of 1050 and 1500 °C, as well as for a sample without the introduction of CTN, the value of ε’ weakly depends on the frequency of the electric field. In the case of samples sintered at 1500 °C, this fact indicates the structural homogeneity of the sample’s low concentration of defects and inclusions. For these samples, low dielectric losses, which are common for polycrystalline alumina ceramics, were observed in the high-frequency range [25]. In the low-frequency region of the spectrum, high losses can be associated with ionic conductivity in a polycrystal, microstructural inhomogeneity (the presence of grain/grain boundary interfaces), as well as the influence of inclusions [26]. In the high-frequency region (1 × 10^3^–1 × 10^5^ Hz), dielectric losses decrease to values of 1 × 10^−4^–3 × 10^−3^. Dielectric losses in this frequency range are related to the internal properties of crystallites. In this region, for a sample with T_s_ = 1050 °C, increased dielectric losses are observed. This can be explained by the presence of a relatively conductive CTN phase, which causes the appearance of polarization at the interfaces [27]. The highest value of ε’, tan δ in the entire frequency range of 2–6 × 10^4^ Hz among sintered pellets is found in a sample with T_s_ = 1300 °C. The low-frequency behavior of tan δ(f) clearly indicates the effect of through conduction [28], while in the frequency range 1 × 10^2^–7 × 10^4^ Hz, a relaxation peak of the volume-charged polarization mechanism is observed due to the presence of the CTN phase. It can also be noted that the value of the permittivity correlates with the shrinkage and density of the sample. Despite this, for a sample with a sintering temperature of 1050 °C, it was found that the presence of quadruple perovskite in the composition of the phase makes it possible to increase the value of the permittivity by a factor of 2 compared to dense corundum ceramics while maintaining low dielectric losses due to the presence of pores.

**Figure 7:**
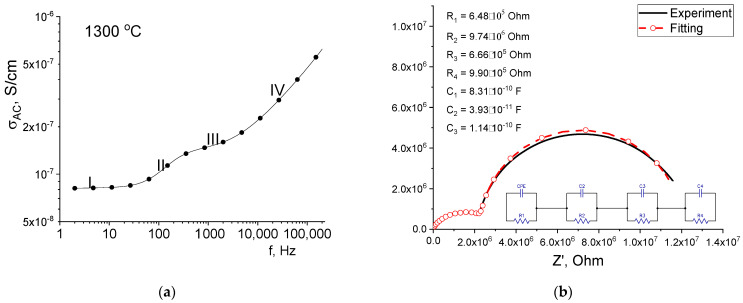
Frequency dependence of AC conductivity (**a**) and Nyquist diagram (**b**) for a sample with T_s_ = 1300 °C.

To explain the frequency behavior of the curves of sample T_s_ = 1300 °C, the frequency dependences of the electrical conductivity on an alternating current were plotted, as well as the Nyquist-Z″(Z′) diagrams. The calculation of *σ_AC_* was done by $\sigma_{AC}=\sigma_{DC}+\varepsilon_{0}\tan\delta \varepsilon^{\prime }2\pi f$, where *DC* conductivity *σ_DC_* was measured as 4.38 × 10^−8^ S/cm. Figure 6a shows the AC conductance spectrum, which shows four regions with different curve slopes (Figure 7a). Different regions can be associated with various conduction mechanisms of electrical current flow. It is obvious that the first mechanism is associated with ionic conduction since no frequency dispersion is observed [29]. In the case of mechanisms II and III, the conductivity is associated with the charge polarization process at the dielectric phase (α-Al_2_O_3_, CuNb_2_O_6_)/conducting phase (CTN) and grain/amorphous grain boundary interfaces. Conduction mechanism IV is associated with the flow of an alternating current in the grains, which is formed when electric charges are oriented along the field while they cannot reach a minimum energy position during a period of sinusoidal voltage. The Nyquist plot is represented by two combined semicircles, which also indicates a pronounced process of dielectric relaxation. To fit the experimental dependence, an equivalent electrical circuit was chosen, including a resistor with a constant phase element and three series-connected parallel RC circuits (Figure 7b). The fitting of parameters R_n_, C_n_ was carried out in the EIS program. According to the results of fitting, in the ceramic sample with T_s_ = 1300 °C, conductive grains with the minimum value of the parameter R_1_ = 6.48 × 10^5^ Ω coexist with insulating grains R_2_ = 9.74 × 10^6^ Ω, as well as grains of the CuNb_2_O_6_ phase and an amorphous grain boundary with parameters R_3_ = 6.66 × 10^5^ Ω and R_4_ = 9.90 × 10^5^ Ω. The charge flow in ceramics with T_s_ = 1300 °C occurs sequentially through the percolation channels formed by the grains of the CTN phase, as well as through the intergranular amorphous phase [30] and non-conducting grains. Thus, a pronounced increase in permittivity is associated with two effects: a decrease in the porosity of ceramics and the formation of percolation (or close to them) electrical channels through grains of the CTN phase. It is worth noting that the use of the CTN additive makes it possible to increase the value of permittivity to 11.55 for alumina ceramics, which is higher than the values reported in other works [2]. In this case, the values of tan δ remain unchanged, which is extremely advantageous when using the obtained ceramics as substrates in microwave electronics.

**Figure 8:**
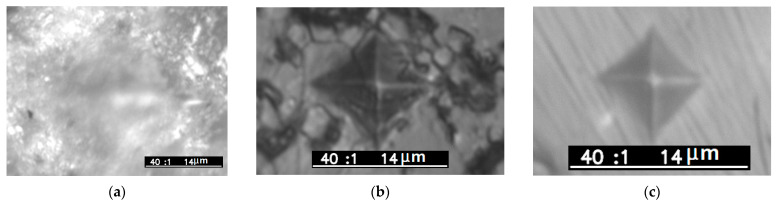
Microphotographs of sample’s surfaces after Vickers indentation test. (**a**) T_s_ = 1050 °C, (**b**) T_s_ = 1300 °C, (**c**) T_s_ = 1500 °C.

Figure 8 shows traces of Vickers indentation from a diamond pyramid. It can be seen that for the 1050 °C sample, a blurred trace is observed due to the high porosity of the ceramic. In the case of a sample with an annealing temperature of 1300 °C, the trace has a classical appearance, but cracks are observed after indentation. This may be due to the stresses that were established by the Williamson–Hall method in the α-Al_2_O_3_ crystal structure and the presence of pores, as well as a wide grain boundary between grains (Figure 3c). The values of HV microhardness for the synthesized samples are lower than those given in other works and the reference literature [2]. In general, with an increase in the sintering temperature, the microhardness increases significantly, which is associated with ceramic densification and an increase in the average grain size [31]. Table 2 compares the results of measurements of microhardness, impedance spectroscopy, and calculation of the average grain size for tablets with different sintering temperatures.

The synthesis of dense α-Al_2_O_3_ ceramics at temperatures T_s_ < 1300 °C is difficult due to the peculiarities of the distribution of the initial components in the pressed green tablets. Since all the powders in this work are almost the same size, there is a possibility of a non-uniform local distribution of the components of the raw mixture. With increasing temperature, the solid-state transformation of copper, niobium, and titanium oxides can occur unevenly in volume, which hinders the formation of the CTN phase, which is responsible for the process of intense densification, according to [21]. In the case of using initial submicron α-Al_2_O_3_ powders and micron powders of other oxides, a different situation should be considered. Firstly, the small sizes of corundum powders sinter more intensively due to the large surface area, which leads to high reactivity. Secondly, local inhomogeneities in the distribution of components occur less often, which facilitates the formation of the CTN phase in ceramics. In the samples studied in this work, intensive densification is possible only at temperatures of complete melting of the 4CuO-TiO_2_-2Nb_2_O_5_ mixture and diffusion of ions through the liquid phase to eliminate the effect of local inhomogeneity in the distribution of components [32]. The results obtained in this work show that the significant effect of reducing the sintering temperature of alumina ceramics is possible only for submicron powders, which can increase production costs. Despite the local uneven distribution of the additive that activates sintering and the presence of pores, the synthesized ceramics show favorable dielectric and mechanical properties that can be in demand in the development of dispersed fuel in the nuclear power industry, the fabrication of ceramic capacitors and substrates for microwave circuits.

## 4. Conclusions

The aid effect of a sintering additive (5 wt.%) with the composition 4CuO-TiO_2_-2Nb_2_O_5_ (CTN) on the dielectric, structural, and mechanical properties of α-Al_2_O_3_ polycrystalline ceramics was investigated. It was demonstrated that introducing CTN sintering aid into the raw powder mixture significantly changes dielectric and mechanical characteristics, even when the micron-sized powder is used as a starting material. The study revealed that samples with the sintering aid showed microhardness HV0.1 values between 1344 and 2263. Using the SEM method, it was found that increasing microhardness and permittivity values occurred due to densification and microstructure changes. These changes are caused by reactive sintering processes and the transition of the CTN additive to the liquid phase during sintering. According to the investigation of XRD patterns, the dominant phase in the synthesized samples is α-Al_2_O_3_, with secondary phases of quadruple perovskite Cu_3.21_Ti_1.16_Nb_2.63_O_12_ and copper niobate CuNb_2_O_6_. The formation of secondary phases significantly increases the permittivity value from 11.6 to 562 in the frequency range of 2–500 Hz, with the quadruple perovskite phase affecting the permittivity most strongly. An explanation of this effect is proposed on the basis of fitting the Nyquist diagram according to the equivalent electrical circuit.

The experimental work has shown that using CTN sintering aid is an effective way to reduce the process temperature in alumina ceramics fabrication in a standard solid-phase route. Furthermore, measured mechanical and dielectric properties indicate the potential multifunctional applicability of the obtained ceramics in the development of dispersed fuels in the nuclear power industry, and the production of ceramic capacitors and substrates for microwave circuits.

## Figures and Tables

**Table 1:** Crystal lattice parameters a and c, mechanical stresses.

Sample	a, nm	c, nm	Strain	Crystalline Size, nm
1500 without additive	0.4759 ± 0.0001	1.2993 ± 0.0001	−0.0004 ± 0.00024	85.6 ± 18
1050	0.4761 ± 0.0001	1.2996 ± 0.0002	−0.0005 ± 0.00031	62.7 ± 17
1300	0.4759 ± 0.0001	1.2996 ± 0.0002	−0.0008 ± 0.00052	84.5 ± 21
1500	0.4759 ± 0.0001	1.2995 ± 0.0002	−0.0008 ± 0.00062	94.8 ± 24

**Table 2:** Comparison of values of microhardness HV0.1, dielectric permittivity, and dielectric loss tangent at frequencies of 100 Hz, 100 kHz, and average grain size d_av_.

Sample	HV0.1	ε’ (100 Hz)	tan δ (100 Hz)	ε’ (100 kHz)	tan δ (100 kHz)	d_av_, μm
1500 without additive	42.6	4.09	0.069	4.09	0.001	2.33
1050	247.5	19.35	0.069	17.88	0.009	2.09
1300	1344.7	562	1.850	15.64	0.475	3.12
1500	2263.5	11.66	0.038	11.55	0.001	10.55

## Data Availability

Not applicable.
